# A Dual-Input Neural Network for Online State-of-Charge Estimation of the Lithium-Ion Battery throughout Its Lifetime

**DOI:** 10.3390/ma15175933

**Published:** 2022-08-27

**Authors:** Cheng Qian, Binghui Xu, Quan Xia, Yi Ren, Dezhen Yang, Zili Wang

**Affiliations:** 1 School of Reliability and Systems Engineering, Beihang University, Beijing 100191, China; 2 School of Aeronautic Science and Engineering, Beihang University, Beijing 100191, China

**Keywords:** lithium-ion battery, SOC estimation, lifetime, recurrent neural network, SOH

## Abstract

Online state-of-charge (SOC) estimation for lithium-ion batteries is one of the most important tasks of the battery management system in ensuring its operation safety and reliability. Due to the advantages of learning the long-term dependencies in between the sequential data, recurrent neural networks (RNNs) have been developed and have shown their superiority over SOC estimation. However, only time-series measurements (e.g., voltage and current) are taken as inputs in these RNNs. Considering that the mapping relationship between the SOC and the time-series measurements evolves along with the battery degradation, there still remains a challenge for RNNs to estimate the SOC accurately throughout the battery’s lifetime. In this paper, a dual-input neural network combining gated recurring unit (GRU) layers and fully connected layers (acronymized as a DIGF network) is developed to overcome the above-mentioned challenge. Its most important characteristic is the adoption of the state of health (SOH) of the battery as the network input, in addition to time-series measurements. According to the experimental data from a batch of LiCoO_2_ batteries, it is validated that the proposed DIGF network is capable of providing more accurate SOC estimations throughout the battery’s lifetime compared to the existing RNN counterparts. Moreover, it also shows greater robustness against different initial SOCs, making it more applicable for online SOC estimations in practical situations. Based on these verification results, it is concluded that the proposed DIGF network is feasible for estimating the battery’s SOC accurately throughout the battery’s lifetime against varying initial SOCs.

## 1. Introduction

Rechargeable lithium-ion batteries have been widely used in electric vehicles (EVs), energy storage systems, etc., due to their merits of a low self-discharge rate, high power density, a long lifespan, etc. [1,2,3,4]. For a steady-state operation of batteries, the online SOC estimation throughout its lifetime plays a key role in the battery management system. On the one hand, from the perspective of user experience, the online SOC estimation of the batteries is an important promise that predicts the remaining driving range of EVs, which is meaningful in reducing the range anxiety of drivers [5,6]. On the other hand, from the perspective of battery management, the online SOC estimation also helps to avoid over-charge and over-discharge of the batteries, thus ensuring a high level of safety and reliability. For these reasons, the online SOC estimation of lithium-ion batteries has been a hot research topic in past decade.

As introduced in the literature [7,8,9], the lithium-ion battery’s SOC estimation approaches are mainly divided into four categories, which include the ampere-hour integral (AHI) methods [10,11,12], the open-circuit voltage (OCV)-based methods [13,14,15], the model-based methods, and the data-driven methods. The former two methods are relatively simple and easy to operate, but at the expense of uncontrollable SOC estimation error. For instance, the AHI methods obtain the battery’s SOC by integrating the battery current over time. However, an accurate initial SOC of battery is hard to obtain in practice as the charge/discharge behavior of the end-users is uncertain. The OCV-based methods are usually applied to estimate the battery’s SOC based on the nonlinear monotonical relationship between the SOC and the OCV. They are, unfortunately, not suitable for online estimation as they require the battery to rest sufficiently to measure its OCV. Moreover, during the operational process, the OCV–SOC curve of the battery shows an obvious shift with the battery state of health (SOH) degradation [16], resulting in a limitation in using the OCV-based methods for batteries over their entire lifetime.

In the model-based methods, models describing the dynamic behavior of LIBs, such as the equivalent circuit model (ECM), the electrochemical model and the electrochemical impedance model are commonly used for SOC estimation. Taking the ECM as an example, circuit elements including capacitors, resistors, and voltage source, etc., are utilized to model the dynamic response between the battery’s terminal voltage and the current. On the basis of those models, advanced filter technologies, such as Kalman filter [17], the extended Kalman filter [18], the unscented Kalman filter [19], the particle filter [20], etc., and observers, such as the sliding mode observer [21], the discrete-time nonlinear observer [22], the extended state observer [23], etc., are frequently utilized to estimate the battery’s SOC. However, the performance of the model-based methods is highly dependent on the accuracy of the battery models, which are still facing challenges in reaching a comprehensive adaptability for different operating conditions.

In contrast to the model-based methods, data-driven methods take the battery as a black box without paying attention to the physical essence of it. These methods estimate the battery’s SOC directly by learning non-linear relationships between the SOC and the battery measurements (such as current and voltage) [24,25,26,27,28,29,30,31,32,33]. In particular, more and more neural networks have been developed for SOC estimation due to their strong ability for nonlinear fitting [34]. Among them, feedforward neural networks were first used for SOC estimation by using voltage, current, and temperature measured at a single step as inputs [27]. Afterwards, RNNs, such as the long short-term memory (LSTM) network [28,29] and the GRU network [30], which take time-series battery measurements as inputs, have also been applied for battery’s SOC estimation. Based on their flexible and powerful capacity in modeling sequential data, the RNNs exhibit high performance in SOC estimation. However, there still exist problems that limit its application over the battery’s entire lifetime. For instance, only time-series measurements (such as current, voltage, and temperature of the battery) are employed for SOC estimation in these RNNs, while the battery’s SOH is ignored. Considering that the mapping relationship between the battery’s SOC and the time-series measurements is dynamically changed during the degradation of the battery’s SOH [35], ignoring the battery’s SOH will diminish the accuracy of the SOC estimation over its lifetime. In order to solve the above issue, a DIGF network was developed by taking both the time-series voltage and current measurements and the battery’s SOH as inputs in this paper.

## 2. Methodology

### 2.1. DIGF Network

**Figure 1:**
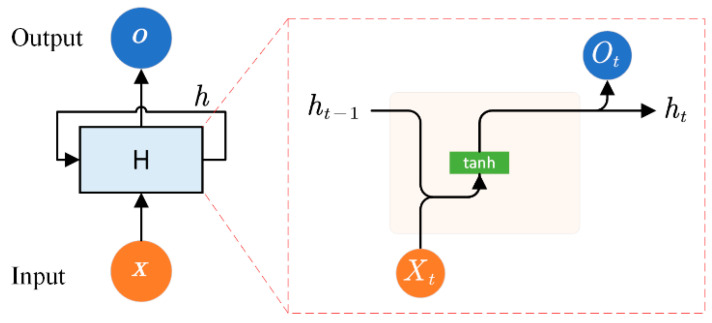
Schematic structure of an RNN.

**Figure 2:**
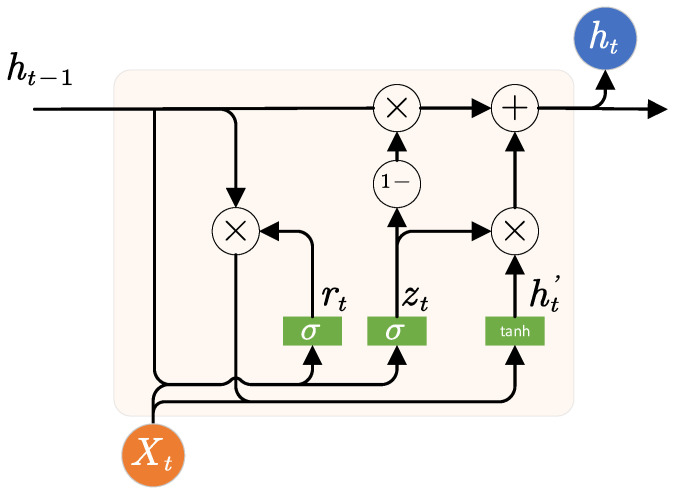
Schematic structure of a GRU cell.

As shown in Figure 1, the outputs of the RNN at the timestep t are related with the inputs $X_{t}$ at that timestep and the outputs $h_{t-1}$ at the previous timestep. This chain-like nature allows the RNN to memorize information from the past, and it is therefore capable of handling sequential data in a large number of applications [36]. However, limited by the vanishing gradient phenomenon in its training process, the traditional RNN can only learn from short-term time-series data [37]. To involve long-term dependencies with a high efficiency, a GRU layer was developed by Cho et al. in 2014 [38]. Generally, the GRU layer can be formulated as:

(1) $$\{\begin{array}{c} z_{t}=\sigma \left(w_{iz}x_{t}+w_{hz}h_{t-1}+b_{z}\right)\\ r_{t}=\sigma \left(w_{ir}x_{t}+w_{hr}h_{t-1}+b_{r}\right)\\ h_{t}^{\prime }=\tanh\left(w_{in}x_{t}+r_{t}*w_{hn}h_{t-1}+b_{n}\right)\\ h_{t}=\left(1-z_{t}\right)*h_{t}^{\prime }+z_{t}*h_{t-1} \end{array}$$

where $X_{t}\in {\mathbb{R}}^{\mu \times 1},\;\;h_{t-1}\in {\mathbb{R}}^{\nu \times 1}$ represent the inputs at timestep t; $h_{t}\in {\mathbb{R}}^{hs\times 1}$ is the output at timestep t; and $z_{t},\;r_{t},\;h_{t}^{\prime }$ are the outputs of the update gate, reset gate, and candidate state, respectively, and $w_{iz}\in {\mathbb{R}}^{hs\times fs}$, $w_{hz}\in {\mathbb{R}}^{hs\times hs}$, $w_{ir}\in {\mathbb{R}}^{hs\times fs}$, $w_{hr}\in {\mathbb{R}}^{hs\times hs}$, $w_{in}\in {\mathbb{R}}^{hs\times fs}$, $w_{hn}\in {\mathbb{R}}^{hs\times hs}$, $\;b_{z}\in {\mathbb{R}}^{hs\times 1}$, $\;b_{r}\in {\mathbb{R}}^{hs\times 1}$, and $b_{n}\in {\mathbb{R}}^{hs\times 1}$ are the parameters of the update gate, reset gate, and candidate state that need to be trained. The μ is the number of features in the input $X_{t}$ and the hyperparameter ν is the number of features in the output $h_{t}$. The schematic structure of the GRU is illustrated in Figure 2. The update gate $z_{t}$ determines the dependencies between the current output $h_{t}$ and the previous output $h_{t-1}$ or candidate state $h_{t}^{\prime }$. Additionally, a small $z_{t}$ close to 0 indicates a high dependency between $h_{t}$ and $h_{t}^{\prime }$. The reset gate $r_{t}$ determines how much the previous output $h_{t-1}$ is used to calculate candidate state $h_{t}^{\prime }$. Those gates help the GRU layer to avoid the problem of gradient vanishing and therefore ensure its capability to learn long-term dependencies from sequential data.

**Figure 3:**
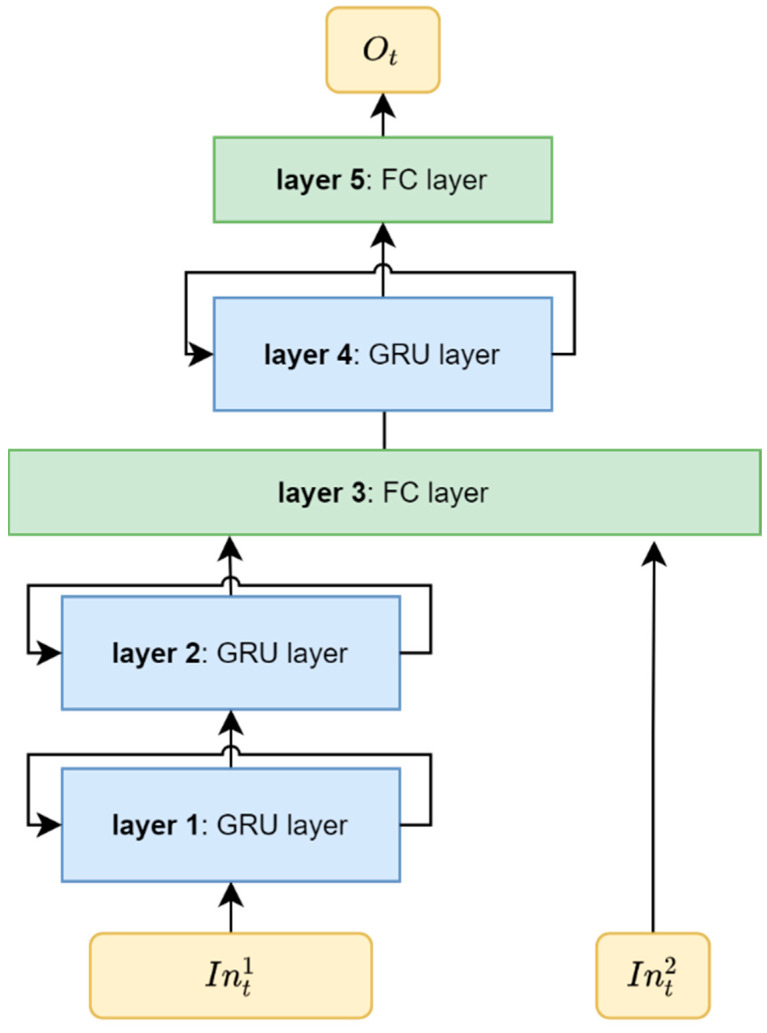
Schematic structure of the proposed DIGF network.

By the merits introduced above, the GRUs are employed in this work to provide SOC estimations. In addition, to take the battery’s SOH into account, a fully connected (FC) layer, as formulated by Equation (2), is added in between the GRU layers to compose a new dual-input neural network (i.e., a DIGF network), as illustrated in Figure 3.

(2) $$out_{fc}=\tan h\left(w_{fc}x_{fc}\right)$$

where $x_{fc}\in {\mathbb{R}}^{\alpha \times 1},\;out_{fc}\in {\mathbb{R}}^{\beta \times 1}$ is the input and output of the FC layer, and $w_{fc}\in {\mathbb{R}}^{\beta \times \alpha }$ are the parameters of the layer that need to be trained. In addition, α is the number of features in the input $x_{fc}$, and hyperparameter β is the number of features in the output $out_{fc}$. Considering that the battery’s SOC is within 1, the hyperbolic tangent function tanh() is employed as the activation function of the FC layer.

As shown in Figure 3, the proposed DIGF network consists of 5 layers in total, including 3 GRU layers (i.e., layers 1, 2, 4) and 2 FC layers (i.e., layers 3, 5). Referring to the sensitivity analysis in [30], the hyperparameters of each layer in the DIGF network are determined and listed in Table 1. The battery’s SOC at timestep t of cycle k is then estimated by two types of inputs, including the measurements ($In_{t}^{1}$) and the battery’s SOH ($In_{t}^{2})$, which are arranged by Equation (3).

(3) $$\{\begin{array}{c} In_{t}^{1}={\left[\begin{array}{c} V_{t}^{k}\\ I_{t}^{k} \end{array}\right]}_{2\times 1}\\ In_{t}^{2}={\left[SOH^{k-1}\right]}_{1\times 1} \end{array}$$

where $V_{t}^{k},\;I_{t}^{k}$ are the voltage and current measurements at timestep t of cycle k, and $SOH^{k-1}$ is the SOH of the battery at cycle k−1. It is noted that, as it is not likely to obtain an accurate $SOH_{k}$ estimation based on few measurements at the beginning of the cycle k, the $SOH^{k-1}$ is employed for the SOC estimation at cycle k as the battery SOHs obtained from two adjacent cycles are quite close. In our study, this $SOH^{k-1}$ value is obtained based on the measurements from the cycle k−1 by an another convolutional neural network model we have developed in [39]. In summary, the proposed DIGF network can be formulated as follows:

(4) $$\{\begin{array}{c} O_{t}^{1}=f_{GRU1}\left(In_{t}^{1},O_{t-1}^{1}\right)\\ O_{t}^{2}=f_{GRU2}\left(O_{t}^{1},O_{t-1}^{2}\right)\\ O_{t}^{3}=f_{FC1}\left(\left[\begin{array}{c} O_{t}^{2}\\ In_{t}^{2} \end{array}\right]\right)\\ O_{t}^{4}=f_{GRU3}\left(O_{t}^{3},O_{t-1}^{4}\right)\\ O_{t}=f_{FC2}\left(O_{t}^{4}\right) \end{array}$$

where $f_{GRU1}(),f_{GRU2}(),f_{GRU3}()$ are the functions of the GRU layers that have been illustrated in Equation (1); $f_{FC1}(),\;f_{FC2}()$. are the functions of the FC layers that have been illustrated in Equation (2); $O_{t}^{1},O_{t}^{2},O_{t}^{3},O_{t}^{4}$ are the outputs of layer 1, layer 2, layer 3, layer 4, respectively; and the $O_{t}$ is the output of the DIGF network at timestep t. 

### 2.2. SOC Estimation Procedure

**Figure 4:**
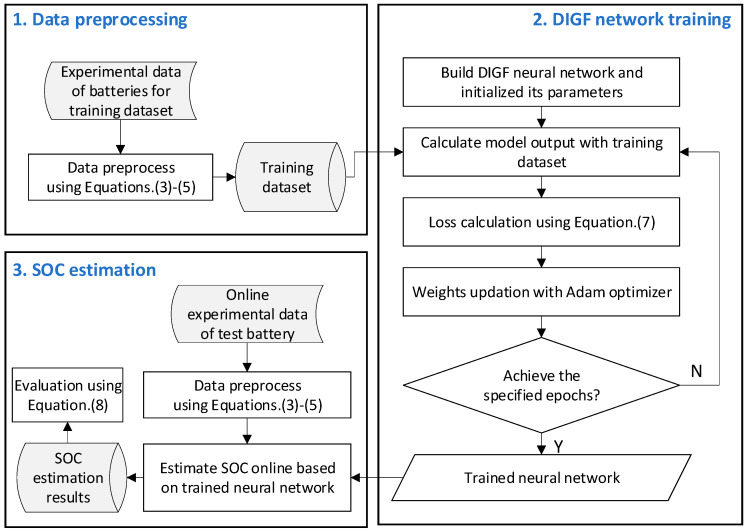
Procedure of SOC estimation of the battery by using the proposed DIGF model.

The procedure for obtaining the SOC estimation by using the proposed DIGF network contains three steps, i.e., data preprocessing, DIGF network training, and SOC estimation, as illustrated in Figure 4.

#### 2.2.1. Data Preprocessing

Firstly, to minimize the effect of different sampling frequencies, the voltage and current measurements are interpolated quadratically in terms of equidistant discharged capacities with an increment of dq. The dq is calculated by Equation (5):

(5) $$dq=\frac{C^{0}}{N}$$

where $C^{0}$ is the battery’s rated capacity, and N is equal to 120 in this paper.

Next, a scaling normalization, shown by Equation (6), is performed on all interpolated voltage and current data to improve the stability of the DIGF network and to speed up the training process as well [40].

(6) $$x_{norm}^{i}=-1+2\times \frac{x^{i}-min\left(x_{train}^{i}\right)}{max\left(x_{train}^{i}\right)-min\left(x_{train}^{i}\right)}$$

where $x_{train}^{i}$ is the unnormalized current/voltage in the training dataset, and $x^{i},\;x_{norm\;}^{i}$ are the unnormalized and normalized discharge voltage/current in the training or testing datasets, respectively.

#### 2.2.2. DIGF Network Training

As the Adam optimizer is capable of handling sparse gradient problems and is easy to implement where the default hyperparameters perform well on most problems [41], it is employed to iteratively update the parameters of the proposed DIGF network in the training process. The formulation of the Adam optimizer is summarized in Equation (7).

(7) $$\{\begin{array}{c} g_{n}=\nabla_{\theta }L_{n}\left(\theta_{n-1}\right)\\ m_{n}=\beta_{1}m_{n-1}+\left(1-\beta_{1}\right)g_{n}\\ v_{n}=\beta_{2}v_{n-1}+\left(1-\beta_{2}\right)g_{n}^{2}\\ {\hat{m}}_{n}=\frac{m_{n}}{1-\beta_{1}^{n}}\\ {\hat{v}}_{n}=\frac{v_{n}}{1-\beta_{2}^{n}}\\ \theta_{n}=\theta_{n-1}-\frac{\eta }{\sqrt{{\hat{v}}_{n}}+\epsilon \;}{\hat{m}}_{n} \end{array}\;$$

where $\theta_{n}$ represents all the parameters of the DIGF network at iteration n; $L_{n}\left(\theta_{n-1}\right)$ is the loss function in terms of $\theta_{n-1}$, calculated by the mean square error (MSE) equation shown in Equation (8); $\beta_{1}$ and $\beta_{2}$ are the decay rates; η is the learning rate; and ϵ is a constant term. The values of $\beta_{1},\;\beta_{2},\;\eta ,\;\epsilon$ are set to 0.9, 0.999, 1 × 10^−3^, 1 × 10^−7^, respectively, according to the literature [41].

(8) $$L_{n}\left(\theta_{n-1}\right)=\frac{1}{M}\overset{M}{\underset{j=1}{\sum }}{\left(SOC_{m,j}-SOC_{e,j}\right)}^{2}$$

in which *M* is the number of samples in the training dataset, and $SOC_{e,j}$, $SOC_{m,j}$ are the experimental and estimated *SOC*, respectively.

#### 2.2.3. SOC Estimation

After the DIGF network is well trained, the online SOC estimation of a new battery can be achieved based on its measurements of the voltage, current, and SOH. Before being imported to the DIGF network, the measured voltage and current data also need to be preprocessed by interpolation and normalization. Then, the root mean square error (*RMSE*) criteria [42] shown in Equation (9) are used to evaluate the standard deviation of the error between the experimental and the estimated SOC values at every cycle, in order to qualify the accuracy of the proposed DIGF network:

(9) $$RMSE=\sqrt{\frac{1}{L}\overset{L}{\underset{j=1}{\sum }}{\left(SOC_{m,j}-SOC_{e,j}\right)}^{2}}$$

where L is the total number of the estimated *SOCs* in each cycle.

## 3. Experimental Data

A batch of test data of LiCoO_2_ batteries, provided by the Center of Advanced Life Cycle Engineering at the University of Maryland, were employed to verify the performance of the proposed DIGF network for SOC estimation throughout the battery’s lifetime [43,44]. These data consist of the test results under room temperature from five batteries (named CS2-33, CS2-34, CS2-35, CS2-36, CS2-37, respectively) with rated capacity of 1.1 Ah. During the experiment, all the batteries were charged under a constant current–constant voltage (CC–CV) charging mode and discharged with a constant current. In a CC–CV charging cycle, the battery is first charged with a constant current until its voltage reaches the maximum charge voltage; then, it is charged under a constant voltage. Then, the constant voltage charging stage is terminated when the charge current tapers down to the end-of-charge current. In the discharging cycle, the battery is discharged with a constant current until its voltage drops to the discharge cut-off voltage. The detailed information on the batteries and the test conditions is listed in Table 2.

**Figure 5:**
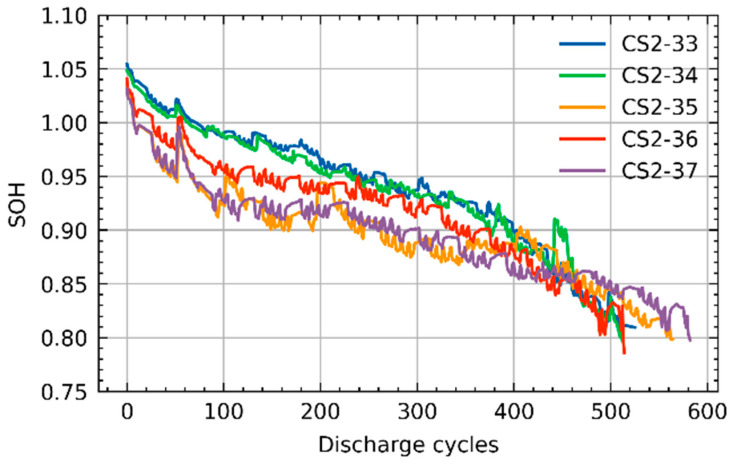
SOH degradation curves of five batteries.

The experimental data of batteries CS2-33, CS2-34, CS2-35, CS2-36 are used for training the proposed DIGF network, whereas the experimental data of CS2-37 are utilized for validation. According to the definition, the benchmark SOC and SOH are calculated by the Equations (10) and (11), respectively.

(10) $$SOC_{t}^{k}=\left(1-\frac{\int_{0}^{t}I_{t}^{k}dt}{\int_{0}^{t_{end}}I_{t}^{k}dt}\right)\times 100\%.$$

(11) $$\{\begin{array}{c} C^{k}=\int_{0}^{t_{end}}I_{t}^{k}dt\\ SOH^{k}=\frac{C^{k}}{C^{0}} \end{array}$$

where $I_{t}^{k}$ is the discharge current at cycle k; $t_{end}$ is the duration of the whole cycle k; $C^{k}$ is the battery capacity at cycle k; $C^{0}$ is the rated capacity of the battery; and $SOC_{t}^{k}$ is the SOC of the battery at time t of cycle k. As the failure threshold of the lithium-ion batteries used in EVs is generally defined as 0.8 of its SOH [45], the lifetime of the battery is determined by the period when its SOH decreases down to 0.8 in this work. As a result, the SOH fading curves before 0.8 of all five of the batteries are shown in Figure 5.

## 4. Results and Discussion

**Figure 6:**
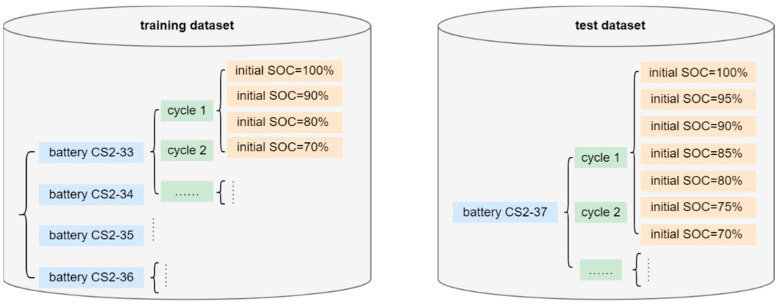
Illustration of the training dataset and test dataset.

For comparison purposes, another two RNN networks (i.e., the LSTM neural network [29] and the GRU neural network [30]) that only take time-series voltage, current, and temperature as inputs are also employed for the SOC estimations. Considering that the batteries in practice are most likely to start discharging with initial SOCs between 70% and 100% [46], the additional dataset consists of discharge cycles with initial SOCs of 90%, 80%, and 70%, which are created by truncating the experimental data of each cycle of the training batteries. Thus, the LSTM, GRU, and DIGF networks are trained with a training dataset that consists of discharge cycle with initial SOCs of 100%, 90%, 80%, and 70%, as shown in Figure 6. Each network is independently trained 10 times, with the consideration of the effects of the random initialization of the network parameters on the SOC estimations.

**Figure 7:**
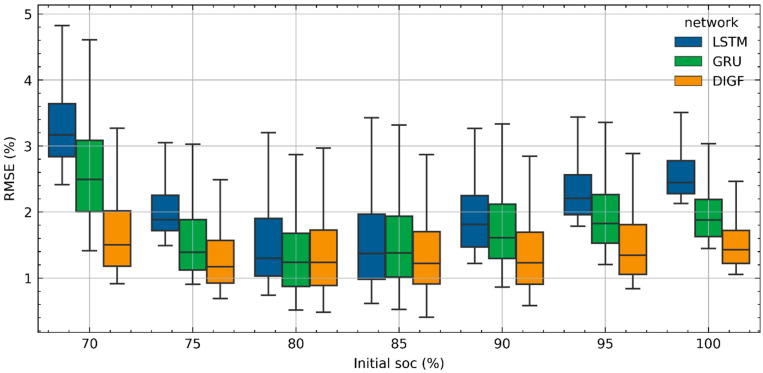
Boxplot of RMSEs of SOC estimations under different initial SOCs for battery CS2-37 throughout its lifetime by LSTM, GRU, and DIGF networks.

For a comprehensive comparison between the performances of the three networks for the SOC estimation throughout the battery’s lifetime, the boxplot of *RMSEs* of the SOC estimations for battery CS2-37 over a lifetime in terms of different initial SOCs are shown in Figure 7. According to Figure 7, the DIGF network achieves the best performance in SOC estimations in both the median and the maximum *RMSEs* in most scenarios, compared to the LSTM and GRU networks. In addition, it also shows a much more stable median *RMSE* of SOC estimations compared to the LSTM and GRU networks, indicating a stronger robustness against the initial SOCs. The above two observations prove the superiority of the DIGF network in the SOC estimations of a battery over its lifetime.

**Figure 8:**
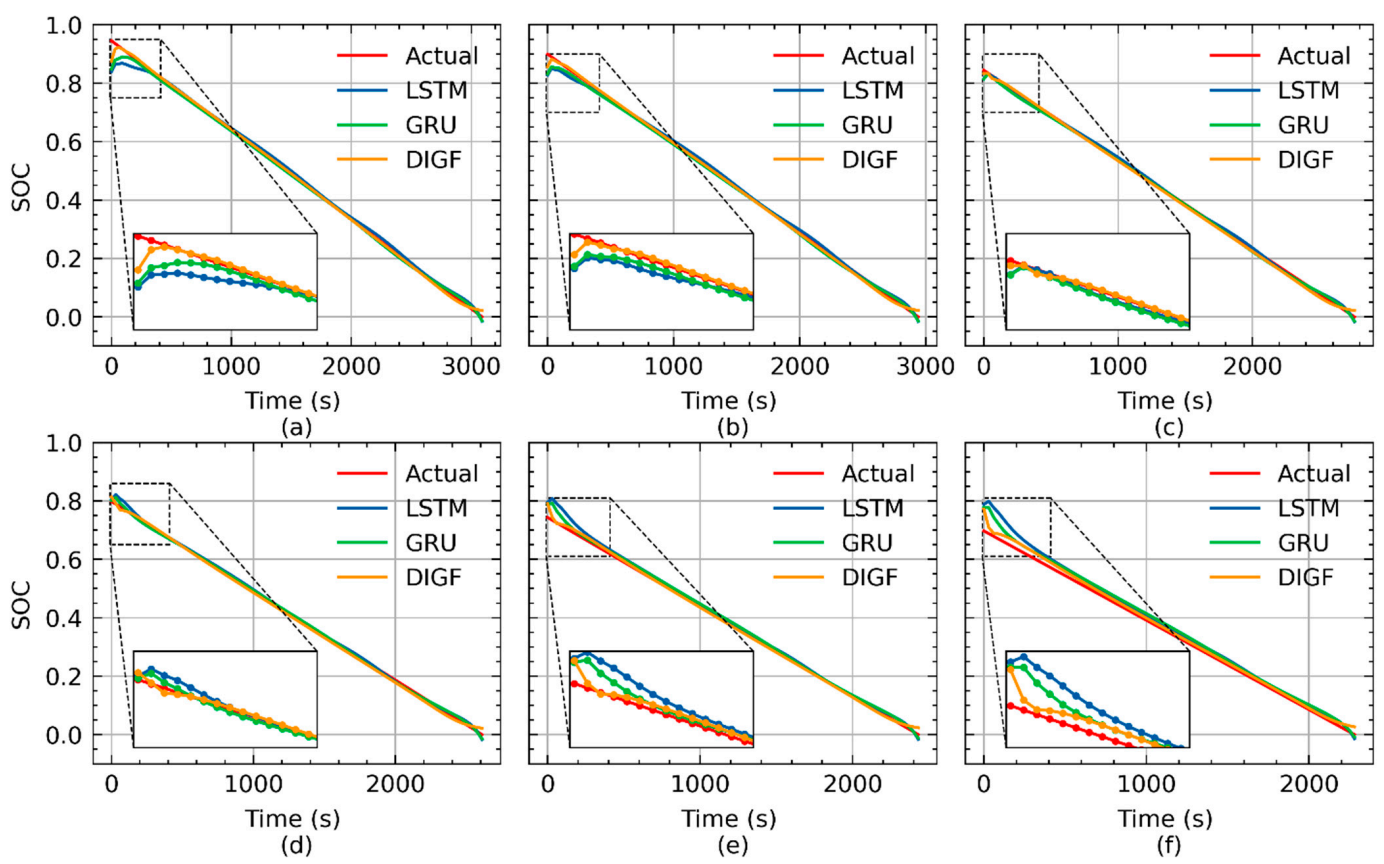
SOC estimations for CS2-37 at cycle 200 by LSTM, GRU, and DIGF networks: (**a**) initial SOC = 95%; **(b**) initial SOC = 90%; (**c**) initial SOC = 85%; (**d**) initial SOC = 80%; (**e**) initial SOC = 75%; (**f**) initial SOC = 70%.

To further explore the performance of the three networks against different initial SOCs, the curves of the SOC estimations for battery CS2-37 under the initial SOCs of 95%, 90%, 85%, 80%, 75%, and 70% at cycle 200 are shown in Figure 8. It can be seen that the large error of the estimated SOCs by the LSTM and GRU networks is majorly concentrated at the first few steps, with all of the initial SOCs from 95% to 70%. As mentioned before, these two networks only take the measurements of the discharge voltages and currents as inputs. However, at the early stage of each discharge cycle, the imported voltage and current data are not sufficient for the LSTM and GRU networks to provide accurate SOC estimations, whereas, owing to the additional input of the SOH, the DIGF network is capable of significantly reducing the SOC estimation error in the early stage of each discharge cycle, and therefore shows stronger robustness against different initial SOCs.

## 5. Conclusions

A DIGF network combining three GRU layers and two FC layers is proposed in this paper for the SOC estimation of lithium batteries over their lifetime. Compared to other RNNs which only take time-series measurements as inputs, the DIGF network employs the battery’s SOH as well as improving the accuracy of the SOC estimations. The experimental results of a batch of LiCoO_2_ batteries show that the proposed DIGF network is feasible for providing satisfying SOC estimations with stronger robustness against different initial SOCs for batteries throughout their lifetimes. Owing to these advantages given above, it is speculated that this proposed DIGF network has great potential for use in online SOC estimation for lithium-ion batteries in practice with a large range of SOHs and initial SOCs.

## Figures and Tables

**Table 1:** Hyperparameters of the DIGF network.

Hyperparameter	Layer 1	Layer 2	Layer 3	Layer 4	Layer 5
*μ*	50	50	-	-	-
*β*	-	-	50	50	1

**Table 2:** Specifications of tested batteries and testing profiles.

Specification	CS2-33	CS2-34	CS2-35	CS2-36	CS2-37
Cell Chemistry	LiCoO_2_ cathode				
Weight (w/o safety circuit)	21.1 g				
Dimensions	5.4 × 33.6 × 50.6 mm				
Rated capacity (Ah)	1.1	1.1	1.1	1.1	1.1
Constant charge current (A)	0.55	0.55	0.55	0.55	0.55
Maximum charge voltage (V)	4.2	4.2	4.2	4.2	4.2
End-of-charge current (A)	0.05	0.05	0.05	0.05	0.05
Discharge cut-off voltage (V)	2.7	2.7	2.7	2.7	2.7
Discharge current (A)	0.55	0.55	1.1	1.1	1.1

## Data Availability

Not applicable.

## Abbreviations

SOC	state-of-charge
RNN	recurrent neural network
GRU	gated recurring unit
FC	fully connected
DIGF	dual-input neural network combining GRU layers and FC layers
SOH	state of health
EV	electric vehicles
AHI	ampere-hour integral
OCV	open-circuit voltage
ECM	equivalent circuit model
LSTM	long short-term memory
RMSE	root mean square error
CC–CV	constant current–constant voltage
T	Timestep
$X_{t}$	input of RNN
$h_{t-1},h_{t}$	output of RNN
μ	number of features in the RNN input
ν	number of features in the RNN output
$z_{t}$	output of update gate
$r_{t}$	output of reset gate
$h_{t}^{\prime }$	candidate state
$w_{iz},w_{hz},b_{z}$	parameters of update gate in GRU
$w_{ir},w_{hr},b_{r}$	parameters of reset gate in GRU
$w_{in},w_{hn},b_{n}$	parameters of candidate state in GRU
σ()	sigmoid function
tanh()	hyperbolic tangent function
$x_{fc}$	input of FC layer
$w_{fc}$	parameters of FC layer
α	number of features in the input of FC layer
β	number of features in the output of FC layer
$out_{fc}$	output of FC layer
k	index of cycle
$V_{t}^{k}$	voltage measurement of battery
$I_{t}^{k}$	current measurements of battery
$In_{t}^{1}$	input 1 of DIGF network
$In_{t}^{2}$	input 2 of DIGF network
$f_{GRU}()$	function of GRU layer in the DIGF network
$f_{FC}()$	function of FC layer in the DIGF network
$O_{t}^{1},O_{t}^{2},O_{t}^{3},O_{t}^{4}$	output of layer 1, layer 2, layer 3, layer 4 in DIGF network
$C^{0}$	battery’s rated capacity
dq	interpolation interval
N	number of interpolation samples
$x_{train}^{i}$	unnormalized current/voltage in the training dataset
$x^{i}$	unnormalized voltage/current in the training or testing datasets
$x_{norm}^{i}$	normalized voltage/current in the training or testing datasets
n	index of iteration during training process
$\theta_{n}$	all parameters of DIGF network
$L_{n}\left(\theta_{n-1}\right)$	loss function
$\beta_{1},\beta_{2}$	decay rates of Adam optimizer
η	learning rate of Adam optimizer
ϵ	constant term of Adam optimizer
M	number of samples in the training dataset
$SOC_{e,j}$	experimental SOC
$SOC_{m,j}$	estimated SOC
L	total number of the estimated SOCs in cycle
$t_{end}$	duration of the discharge cycle
$C^{k}$	battery capacity at cycle k
$SOC_{t}^{k}$	battery SOC at time t of cycle k
$SOH^{k}$	battery SOH at cycle k
